# Hemodynamic Comparison of Two Nasal Endotracheal Intubation Techniques Using Fiberoptic and Conventional Approaches in Jaw Fracture Surgery Patients: A Study from Kerman, Iran (2020): Nasal Endotracheal Intubation Techniques in Jaw Fracture Surgery Patients

**DOI:** 10.31661/gmj.v13iSP1.3626

**Published:** 2024-12-25

**Authors:** Naeimeh Naeimi Bafghi, Shirin Salagegheh, Jafar Salehi, Neda Naeimi Bafghi, Mahdieh Tajaddini

**Affiliations:** ^1^ Clinical Research Center Shahid Bahonar Hospital Kerman University of Medical, Sciences Kerman, Iran; ^2^ Clinical Research Center Shafa Hospital Kerman University Of Medical Sciences, Kerman, Iran

**Keywords:** Changes, Endotracheal Intubation, Fiberoptic Bronchoscope, Maxillofacial Surgery, Blood Pressure, Anesthesia

## Abstract

**Background:**

Laryngoscopy and endotracheal intubation can cause significant hemodynamic changes, resulting in potentially life-threatening complications. This study aimed to compare the hemodynamic effects of traditional endotracheal tube and fiberoptic bronchoscope methods in patients undergoing maxillofacial fracture surgery.

**Materials and Methods:**

In this randomized clinical trial, 60 patients with jaw fractures at an institutional tertiary hospital in Kerman, Iran, in 2020 were enrolled. Participants were randomly assigned to either the traditional endotracheal tube group (n=30) or the fiberoptic bronchoscope group (n=30). Hemodynamic parameters, including systolic and diastolic blood pressure, heart rate, and arterial oxygen saturation (SpO2), were recorded at baseline, and 1, 3, 5, 10, and 15 minutes after anesthesia. Additional assessments included time to peak blood pressure, frequency of SpO2 drop, and postoperative nausea, vomiting, and hoarseness.

**Results:**

There were significant differences in systolic, diastolic, and mean arterial blood pressures, as well as heart rate, between the two groups (p 0.05). The highest hemodynamic values occurred 3 minutes after anesthesia in the traditional intubation group and immediately after intubation in the fiberoptic group. SpO2 levels remained stable at 99% throughout the study in both groups. Hemodynamic values declined by the 10th and 15th minutes post-anesthesia. The study reveals that fiberoptic intubation is associated with lower early hemodynamic fluctuations compared to traditional intubation. This is particularly important as it suggests that fiberoptic intubation may mitigate the risk of cardiovascular complications, which can be life-threatening in vulnerable patient populations.

**Conclusion:**

Fiberoptic intubation resulted in lower early hemodynamic fluctuations compared to the traditional method, suggesting it may be a safer option for reducing cardiovascular stress during maxillofacial surgery. The current article presents a novel comparison of hemodynamic effects between traditional endotracheal intubation and fiberoptic bronchoscope methods specifically in the context of maxillofacial fracture surgery.

## Introduction

Endotracheal intubation is the procedure of placing a flexible plastic tube into the trachea to establish an airway, which allows for mechanical ventilation. The most common method of intubation is through the oral route, using a laryngoscope. This procedure is typically performed when a patient requires mechanical breathing or to prevent aspiration [1]. Endotracheal intubation is commonly assisted by direct laryngoscopy during general anesthesia to prevent pulmonary aspiration [2]. While intubation is typically performed orally, in certain cases, such as during mouth surgery or in patients needing prolonged intubation in intensive care unit (ICU) setting, nasal intubation is preferred [3]. Nasal tracheal intubation (NTI) is often regarded as the procedure of choice in head and neck surgeries [4], since it allows the surgeon greater access to the surgical site, which may improve the procedure outcome. Additionally, NTI enables the surgical team to manage potential anesthesia-related complications more efficiently [3].

Despite many advantages, NTI is associated with certain risks, and complications can be serious, contributing to anesthesia-related deaths [5][6]. Potential complications include epistaxis [7], bacteremia [8], and partial or complete airway obstruction [9]. Other risks include submucosal laceration [10] and perforation [11], as well as retropharyngeal perforation [12]. Several factors contribute to NTI complications, which can be classified into three categories: a) patient factors; such as unfavorable anatomy, craniofacial abnormalities, or pre-existing conditions like gastroesophageal reflux; b) tube-related factors; including using an excessively long tube, high cuff pressure, or the presence of a nasogastric tube; and c) technical factors; such as forceful intubation, poor laryngeal visibility, and multiple intubation attempts [13].

Another common method for intubation is based on fiberoptic bronchoscopy. This technique is particularly useful for patients who are anticipated to have difficult intubations [1]. However, fiberoptic intubation is costly and time-demanding, and requires specialized skills. While direct laryngoscopy can be used to complete intubation in less than 20 seconds, fiberoptic intubation is considerably limited in emergency settings due to the longer time required [4][14]. Nevertheless, fiberoptic intubation is still a valuable technique for establishing a secure airway in certain situations. For instance, it is beneficial when the neck cannot be moved due to vertebral injury or when the vocal cords cannot be directly visualized via laryngoscopy. In such cases, fiberoptic intubation can be performed either orally or nasally, under general anesthesia or even while the patient is awake [15].

As is the case with many semi-invasive procedures, intubation confers risks. Complications during endotracheal intubation may include trauma, laryngospasm, bronchospasm, cardiac arrhythmias, incorrect tube placement in the esophagus, over-insertion of the tube, vomiting, potential aspiration, hypoxia due to delays, and upper airway trauma [1]. Sympathetic stimulation [16] and hemodynamic changes, such as alterations in heart rate and blood pressure, are also common concerns for anesthesiologists, who aim to avoid these issues [17][18]. Whether intubation is performed directly or via fiberoptic, the physical stress and pain caused by laryngoscopy and nasal intubation can lead to significant changes in patient hemodynamics [17][19]. These changes can include elevated blood pressure and heart rate, which could be life-threatening in patients with pre-existing hypertension (20), coronary artery disease, valve disorders, or cardiac tamponade [17][20]. Additionally, these hemodynamic shifts can be hazardous for patients with intracranial complications [21][22].

Maintaining patient safety and preventing complications during laryngoscopy and intubation are pressing matters, as failure meet such prerequisites can lead to unfavorable shifts in hemodynamics [23]. Laryngoscopy and tracheal intubation are often accompanied by such changes. One suggested method to reduce these effects is the use of nasal intubation with a fiberoptic bronchoscope [24]. To test the veracity of this hypothesis, this study aims to compare two fiberoptic intubation techniques (tracheal tube first vs. fiberoptic scope first) in patients undergoing maxillofacial fracture surgery at an institutional tertiary hospital in Kerman, southern Iran, in 2020.

## Materials and Methods

### Study design

This the present work was randomized clinical trial consisting of two interventional arms, including ‘tracheal tube first’ and ‘fiberoptic scope first’ involving 60 patients undergoing maxillofacial surgery.

### Study population

The study population consisted of patients aged 16 to 65, with American Society of Anesthesiologists (ASA) physical status classification I or II, who were candidates for maxillofacial fracture surgery at an institutional tertiary hospital in Kerman, Iran, in 2020. Patients were recruited to the study using convenience sampling based on a set of predefined inclusion and exclusion criteria.

### Sample size and calculation

The sample size was calculated based on the work of Farbood et al. [24], which reported mean diastolic blood pressures (DBP) of

98.7 ± 9.39 and 94.2 ± 6.27 mmHg in two groups of 50 patients 4 minutes after the onset of intubation. Assuming an alpha (significance) value of 0.05 and a power of 0.80, the final sample size for each group of the current study was determined to be 30 for each interventional group, resulting in a total sample size of 60. The power of 0.80 was determined to be appropriate based on standard conventions in clinical trials, indicating an 80% chance of detecting a true effect if it exists. This power level is typically considered sufficient to minimize the risk of Type II errors. Both groups experienced similar rates of these complications, suggesting that the choice of intubation technique did not adversely affect postoperative recovery. Detailed statistics on the frequency of these outcomes in each group can be provided to support this conclusion.

### Patient eligibility criteria

Patients meeting specific inclusion criteria were considered as potentially eligible, until the presence of certain exclusion criteria were ruled out. Our inclusion criteria were as follows: ASA class I and II, Age between 16 and 65, and Candidate for maxillofacial surgery following jaw fracture.

Patients were excluded if they had any of the following conditions: Difficult airway anatomy, Concurrent skull or mid-face fractures, Nasal or sinus infections, Chronic diseases (e.g., hypertension, diabetes, thyroid disorders, neuropathies, multiple sclerosis), disorders of the spinal cord or autonomic nervous system (ANS), History of head trauma, brain surgery or seizures, Pulmonary diseases, and Gastroesophageal reflux.

### Study procedure

A total of 60 patients with jaw fractures who met the inclusion criteria were enrolled. If a patient was under treatment for hypertension, they were instructed to discontinue their antihypertensive medications at least 10 days before surgery. Patients were randomly assigned into two groups (N=30) using block randomization method, with each block consisting of four patients (N=4) undergoing either ‘tracheal tube intubation first’ (TTI) or ‘fiberoptic intubation first’ (FOI) in random orders of permutations determined using a randomizer software. The randomization process was conducted using a block randomization method, where patients were divided into blocks of four. Each block contained a random permutation of the two intervention groups. This method ensured that the assignment of patients to each group was concealed and unbiased, as the randomization was performed using a randomizer software that generated the order of assignments prior to patient enrollment.

The monitoring protocols included the use of electrocardiography (ECG) for heart rate, a non-invasive blood pressure monitor for blood pressure measurements, and pulse oximetry for arterial oxygen saturation (SpO2). These devices are standard in clinical practice and have been validated through extensive clinical use and research. Calibration of the blood pressure monitor was performed before the study, and regular checks were conducted to ensure the accuracy of the readings throughout the procedure. Upon arrival at the operating room, hemodynamic monitoring tools including ECG, pulse oximeter, non-invasive blood pressure monitor were attached, and baseline systolic and diastolic blood pressure (SBP/DBP) and heart rate (HR) were recorded. Anesthesia was induced similarly in both groups by the same anesthesiologist. Pre-oxygenation was performed for 2 minutes, followed by combined administration of midazolam (0.05 mg/kg) and fentanyl (2 µg/kg) for sedation. Three minutes later, pre-intubation blood pressure and heart rate were recorded.

In the TTI group, intubation was performed using a fiberoptic bronchoscope and nasal tracheal tube by a third-year anesthesiology assistant. Lidocaine (0.5-1 mg/kg) was sprayed on the vocal cords through the bronchoscope during visualization. Male patients were intubated with a nasal tube size 7-7.5, while female patients were intubated with a nasal tube size 6.5-7. In the FOI group, fiberoptic intubation was performed first using a 5 mm diameter bronchoscope through the nasal passage by the same anesthesiology assistant. Similarly, tracheal tubes were size 7-7.5 for male and 6.5-7 for female patients. The number of unsuccessful attempts was recorded in both groups. If the time required for successful intubation exceeded 120 seconds, the patient would be excluded from the study.

Immediately after intubation, anesthesia was induced with thiopental (5 mg/kg) and atracurium (0.5 mg/kg). Maintenance anesthesia was provided using a 1:1 mixture of O2 and N2O with 1-1.5% isoflurane. SBP, DBP and HR were measured at 1, 3, 5, 10, and 15 minutes after anesthesia induction. Time to peak blood pressure and the number of SpO2 drops were also recorded. After intubation, patients were ventilated with a volume of 10 mL/kg, and end-tidal CO2 was recorded after five breaths. Postoperative nausea, vomiting, and hoarseness were monitored in the recovery room. This study found no significant differences in postoperative outcomes, including nausea, vomiting, or hoarseness, between the two intubation methods. These outcomes were assessed by recovery room staff who monitored patients for these complications after surgery. The frequency of each outcome was recorded, and statistical analysis was performed to compare the rates between the two groups, confirming that both techniques resulted in similar postoperative experiences for patients.

### Data collection tools

The following tools were used to collect patient data: A checklist for recording patient data, a Pulse oximeter for arterial oxygen saturation monitoring, an Arm blood pressure monitor, a Laryngoscope, a Fiberoptic bronchoscope.

### Ethical considerations

The protocol of this study was approved by the institutional Research Ethics Committee (Approval ID: IR.KMU.AH.REC.1399.023). Prior to participation, the patients were asked to provide their written informed consent, once the procedures of the study were explained to them. The trial was registered with the Iranian Registry of Clinical Trials (IRCT) (Registration No.: IRCT20210301026866N7).

### Statistical analysis

The collected data were statistically analyzed using IBM SPSS software version 22 (IBM Corp., Armonk, N.Y., USA). Qualitative and quantitate statistics were presented as frequency and mean ± standard deviation (SD). Repeated measures ANOVA and Friedman tests were used to analyze mean ± SD across different time-points between the two groups for variables with normal and non-normal distribution, respectively, with P < 0.05 being considered statistically significant.

## Results

**Figure-1:**
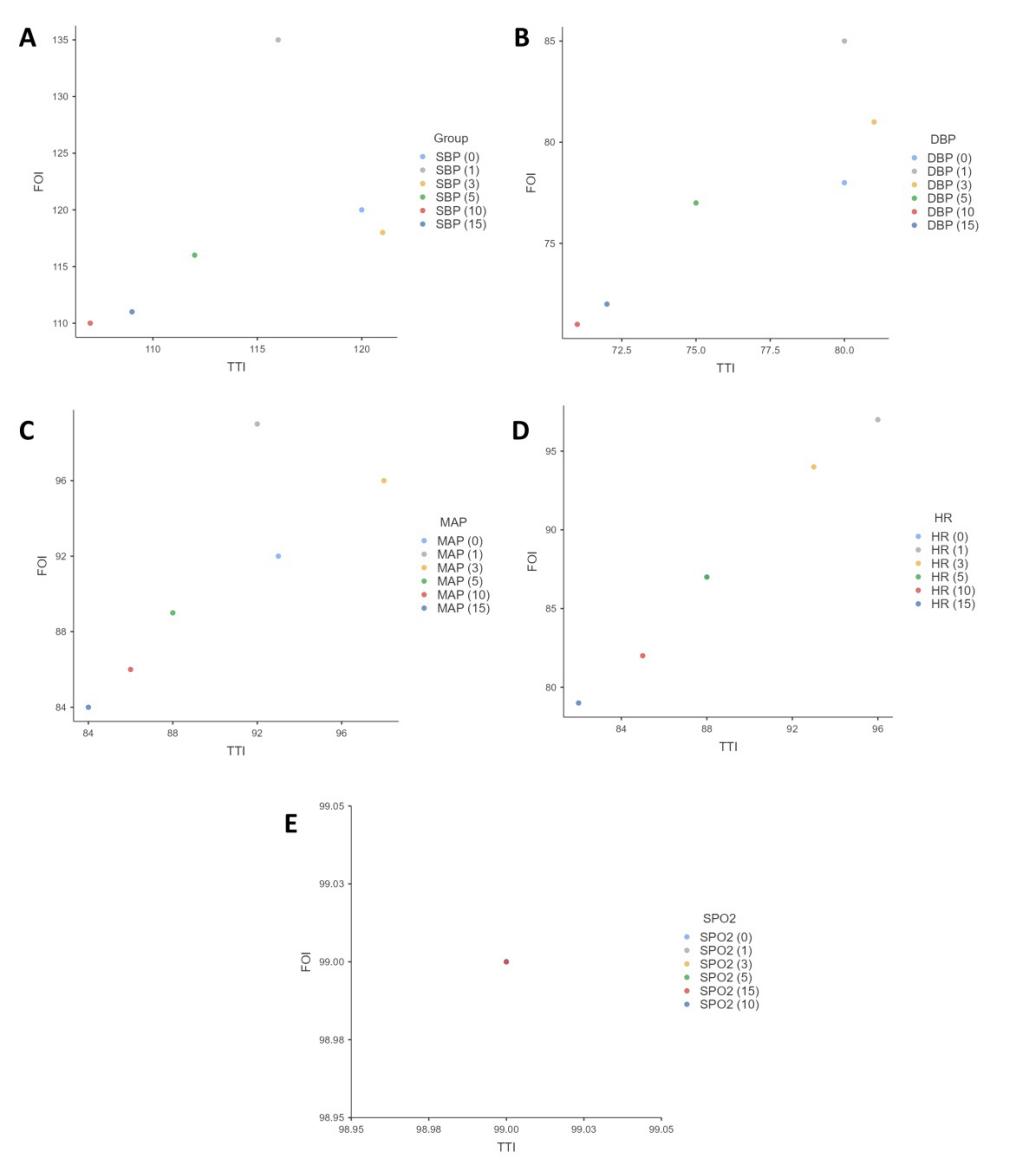
Figure 1. Scatter plots of average systolic blood pressure (A), diastolic blood pressure (B), mean arterial pressure (C), heart rate (D) and SpO2 (E) across different time-points in tracheal tube intubation (TTI) and fiberoptic intubation (FOI) groups.

**Table:** Table1. Comparison of mean age and weight between groups.

Demographic	Group		P-value
	TTI (N=30)	FOI (N=30)	
Age (yr)	33.33 ± 2.53	29.83 ± 2.14	< 0.001
Weight (kg)	62.60 ± 1.72	63.63 ± 1.24	0.010

**Table:** Table2. Comparison of mean systolic blood pressures (SBP) across different time-points between groups.

**Time-Point (min)**	**Group (N=60)**		**P-value**
	**TTI (N=30)**	**FOI (N=30)**	
Baseline	120	120	
1	116	125	
3	121	118	< 0.001
5	112	116	
10	107	110	
15	109	111	

P < 0.05 is considered statistically significant

**Table:** Table3 Comparison of mean diastolic blood pressures (DBP) across different time-points between groups.

**Time-Point (min)**	**Group (N=60)**		**P-value**
	**TTI (N=30)**	**FOI (N=30)**	
Baseline	80	78	
1	80	85	
3	81	81	< 0.001
5	75	77	
10	71	71	
15	72	72	

P < 0.05 is considered statistically significant

**Table:** Table4 Comparison of average mean arterial pressure (MAP) across different time-points between groups.

**Time-Point (min)**	**Group (N=60)**		**P-value**
	**TTI (N=30)**	**FOI (N=30)**	
Baseline	93	92	
1	92	99	
3	98	96	< 0.001
5	88	90	
10	86	86	
15	84	84	

P < 0.05 is considered statistically significant

A total of 60 patients, X men and Y women, with a mean age and weight of 31.58 ± 2.91 years and 63.11 ± 1.57 kg, respectively, were recruited. All intubations were performed successfully, as such, none of the initially included patients were excluded. As indicated in Table-1, Patients in the ‘tracheal tube intubation first’ (TTI) group were significantly older compared to the ‘fiberoptic intubation first’ (FOI) group. Conversely, the mean weight of patients in the FOI group was significantly higher than that of the TTI group (P=0.010).

P < 0.05 is considered statistically significant. Primary clinical parameters that were used for comparison of time-specific hemodynamic status between the TTI and FOI groups included systolic blood pressure (SBP), diastolic blood pressure (SBP), mean arterial blood pressure (MAP), heart rate (HR) and SpO2, the scatter plots of which are visualized in Figure-1. Systolic blood pressures were recorded at 0, 1-, 3-, 10- and 15-minutes post-anesthesia. Table-2 summarizes the mean SBPs at different time-points for the two intervention groups. The highest mean SBPs were 121 and 125 mmHg, which were recorded in minutes 3 and 1 for TTI and FOI groups, respectively. Despite the equal baseline values, SBPs were different between the two groups, which was deemed as statistically significant (P < 0.001).

The mean diastolic blood pressures (DBP) in different time-points for the two intervention groups are listed in Table-3. The lowest DBPs in the TTI and FOI groups were recorded at 10 minutes post-anesthesia, respectively, which were equal to each other (71 mmHg). Conversely, the highest DBP were 81 and 85 mmHg, which were recorded at 3- and 1-minute post-anesthesia in TTI and FOI groups, respectively. Nevertheless, the initial difference in DBP values between the two group were found to be statistically significant (P<0.001).

Table-4 lists the average values of mean arterial pressure (MAP) at different time-points in the two groups. As can be seen, there was a statistically significant difference between the two groups until minute 10 (P < 0.001). The highest MAPs, being 98 and 99 mmHg, were recorded at minute 3 and minute 1 post-anesthesia in TTI and FOI groups.

The mean hear rates (HR) at different time-points are reported in Table-5 for both groups. The highest mean HRs, 96 bpm in TTI and 97 bpm in FOI, were both recorded at 1-minute post-anesthesia. Similar to other parameters, the difference between the two groups with regard to HR was found to be statistically significant (P < 0.001), with the TTI group showing relatively higher end-point HRs.

Comparison of mean SpO2 levels between the two groups at any time-point revealed no statistically significant difference (P=0.940). Importantly, SpO2 levels were maintained at 99% in both groups across all time-points, indicating minimal disturbance in SpO2 balance following intubation.

As shown in Table-6, the mean operation time in the TTI group was significantly higher to that of the FOI group (161.0 ± 9.26 min vs. 148.0 ± 8.74 min). Additionally, we also observed a marked difference between intubation times, with the TTI group showing significantly higher intubation time compared with the FOI group (95.0 ± 4.10 s vs.

91.0 ± 5.47 s), indicating that fiberoptic intubation was associated with better perioperative outcomes.

Lastly, none of the patients exhibited postoperative nausea, vomiting or agitation, indicating a high level of tolerance among patients.

## Discussion

**Table:** Table5. Comparison of mean heart rate (HR) across different time-points between groups.

Time-Point (min)	Group (N=60)		P-value
	TTI (N=30)	FOI (N=30)	
Baseline	88	87	
1	96	97	
3	93	94	< 0.001
5	88	87	
10	85	82	
15	82	79	

P < 0.05 is considered statistically significant

**Table:** Table6. Comparison of mean operation and intubation times across different time-points between groups.

Variable	Group (N=60)	P-value
TTI (N=30)	FOI (N=30)
Operation Time (min)	161.0 ± 9.26	148.0 ± 8.74	< 0.001
Intubation Time (sec)	95.0 ± 4.10	91.0 ± 5.47	0.002

P < 0.05 is considered statistically significant

In this study comparing hemodynamic outcomes between fiberoptic and conventional nasal endotracheal intubation techniques in jaw fracture surgery patients, significant differences were observed. Patients in the fiberoptic intubation group (FOI) had higher systolic, diastolic, and mean arterial pressures at multiple time points compared to the conventional tube-first group (FOI), with all differences being statistically significant

(P < 0.001). In contrast, heart rates were generally higher in the TTI group, while SpO2 levels remained stable and comparable in both groups. In spite of these differences, however, the hemodynamic profile of both groups was acceptable during surgery. Additionally, the FOI group demonstrated shorter intubation and operation times, suggesting it may offer more favorable perioperative outcomes.

Throughout the last 30 years, a limited number of investigations have sought to explore the effects of tube-first and fiberoptic-first intubation on hemodynamic status of patients undergoing surgery. The present study comparing fiberoptic-first (FOI) and tube-first (TTI) nasal intubation techniques in patients undergoing jaw fracture surgery demonstrates significant hemodynamic differences, within the acceptable hemodynamic range, between the two groups. In 2023, Shoukry et al. recruited 40 patients with difficult airway, assigned to two groups of 20 participants, aiming to compare the effects of classic and fiberoptic-first intubation on hemodynamic profile [25]. Similar to our study, Shoukry et al. observed shorter intubation time in the fiberoptic group compared to the classic group (64.25 ± 8.28 vs. 88.25±5.49). They also did not report any significant differences in SpO2 values between the two groups [25], which falls in agreement to our findings. Their observations regarding the hemodynamic status of patients align partially with results reported in the present work. Similar to our work, Shoukry et al. noted significantly lower heart rates in the fiberoptic group. However, they also noticed significantly lower arterial pressure among patients undergoing fiberoptic intubation [25], which delineates from the findings of the present study, in which we observed significantly different but alternating values of MAP between the two groups, that did not fully support the superiority of FOI.

In 2018, Ozkan et al. also found that fiberoptic intubation was associated with fewer hemodynamic disturbances compared to direct laryngoscopy in children undergoing nasotracheal intubation (NTI) [26]. Our study aligns, to some extent, with these results, where FOI resulted in occasionally lower systolic and comparably similar diastolic pressures compared to TTI, suggesting a relatively stable perioperative course, within the acceptable hemodynamic range. Moreover, the higher heart rates observed in the TTI group build upon the findings of Ozkan et al. [26] regarding increased heart rates in the direct laryngoscopy group, further highlighting the benefit of fiberoptic techniques in managing hemodynamic stress during intubation.

Similarly, Aqil et al. (2014) and Aghdaii et al. (2010) found no significant difference in hemodynamic stress responses between fiberoptic and other intubation techniques [27][28]. While our results showed statistically significant time-specific differences in blood pressure and heart rate between the TTI and FOI groups, the end-point hemodynamic parameters between the two groups were similar, supporting the observations of the studies in question, whose different patient populations (normal airways and CABG patients) suggest that the clinical setting and patient condition may influence these outcomes [29][30]. This underscores the need for context-specific evaluation of intubation techniques.

The findings of the current study are consistent with those of previous studies conducted in Iran, particularly in terms of hemodynamic responses to intubation. Mortazavi et al. (2002) demonstrated significant increases in blood pressure and heart rate following laryngoscopy and intubation, with nasotracheal intubation resulting in more pronounced hemodynamic changes than orotracheal intubation [23][31][32]. Similar to the current study, Mortazavi’s results underline the importance of careful monitoring of blood pressure and heart rate during intubation to mitigate the potential complications, especially in patients at risk for cardiovascular events [23][33][34][35][36]. Our findings also highlight the hemodynamic fluctuations observed after intubation in both tube-first and fiberoptic-first groups, reinforcing the relevance of these concerns in clinical practice.

Naghibi et al. (2016) further examined the hemodynamic variations induced by intubation methods, comparing video laryngoscopy and direct Macintosh laryngoscopy in elderly patients. Their results showed that video laryngoscopy led to greater increases in systolic and diastolic blood pressures than Macintosh laryngoscopy, a finding that partially resembles the hemodynamic responses observed in our study, where changes were noted across both groups. While our patient cohort differed in age and intubation technique, both studies emphasize the potential for significant hemodynamic alterations with different intubation approaches, highlighting the necessity of individualized patient management during anesthesia [29][37][38][39]. Future studies can provide deeper insights into the hemodynamic implications of nasal endotracheal intubation techniques in patients undergoing jaw fracture surgery, ultimately improving patient outcomes and safety in anesthetic practice [40][41][42]. Taken together, these studies support the present findings, emphasizing the universal concern of managing intubation-induced hemodynamic changes in clinical settings.

## Conclusion

In conclusion, this study demonstrates that both tube-first and fiberoptic-first intubation techniques induce notable hemodynamic changes in patients with ASA class I or II undergoing maxillofacial surgery. While both approaches resulted in fluctuations in blood pressure and heart rate post-intubation, no significant advantage was observed in terms of hemodynamic stability between the two groups. These findings align with prior research, both internationally and within Iran, indicating the need for careful hemodynamic monitoring during intubation to prevent potential complications, especially in vulnerable patients. The authors believe that further investigation is warranted into the long-term hemodynamic effects of both intubation techniques, particularly in patients with pre-existing cardiovascular conditions. Future studies could be designed to include a larger and more diverse patient population, stratifying participants based on their medical history. Additionally, incorporating a multi-center approach could enhance the generalizability of the findings. Longitudinal studies assessing postoperative recovery and complications over an extended period would also provide valuable insights. Further studies are recommended to explore the long-term clinical outcomes associated with these techniques and to assess their effectiveness in different patient populations.

## Conflict of interest

None.
